# Supplementary material for: Longitudinal Prospective Study of Emergency Medicine Provider Wellness Across Ten Academic and Community Hospitals During the Initial Surge of the COVID-19 Pandemic
Source: Res Sq. 2020 Oct 15:rs.3.rs-87786. Preprint. [Version 1] doi: 10.21203/rs.3.rs-87786/v1 (PMC7574355; doi:10.21203/rs.3.rs-87786/v1)
Supplement: Supplement [file 714aa8eba69cefa2a7a49251.docx]

| **Supplemental Table 1: Timeline of Selected Events and Wellness Initiatives for EM Providers Across During the Study Period** | |
| --- | --- |
| **Date** | **Event** |
| 12-31-19 | 1^st^ COVID-19 cases in China |
| 1-21-20 | 1^st^ COVID019 case person-to-person spread reported in US |
| 1-30-20 | WHO declares Global Health Emergency |
| 3-5-20 | IUH fit testing for N95 prioritized for frontline providers |
| 3-5-20 | IUH healthcare system business travel restriction outside Indiana |
| 3-6-20 | Indiana confirms 1^st^ COVID positive case; Indiana State Health Department CMO webcast briefing |
| 3-10-20 | DEM Work Force Back up Plan and EM COVID Preparedness Plan presented |
| 3-10-20 | DEM educational didactics moved to virtual platform |
| 3-11-20 | DEM physicians establish double back-up call schedule; disaster privileges to cover all ED sites; EM admin work from home |
| 3-11-20 | IUH & IUHP Incident Command announces daily communications |
| 3-12-20 | DEM department childcare exchange launched |
| 3-15-20 | DEM Incident Command adds Wellness Taskforce |
| 3-19-20 | EM COVID-19 wellness resources (mental health, spiritual health, childcare), blogs, morale boosters launched weekly |
| 3-23-20 | Indiana Governor stay-at-home order |
| 3-20-20 | IUH starts COVID testing |
| 3-27-20 | Hospital COVID-19 resuscitation response teams launched to minimize risk & conserve PPE |
| 4-6-20 | DEM secured hospital provided scrubs and showers for EM providers |
| 4-9-20 | PPE mask resterilization procedures established for hospitals |
| 4-13-20 | IUH establishes next-day virtual primary care follow up for COVID suspected ED patients |
| 4-15-20 | Dept of Psychiatry announces system wide mental health support program |
| 4-21-20 | DEM Wellness Taskforce promotes sense of unity with #EMfrontline logo, slogan, and challenge coins |
| 4-24-20 | DEM providers offered COVID-19 serology testing (IgG) |

EM = Emergency Medicine

COVID = coronavirus disease; COVID-19; SARS-CoV-2

WHO = World Health Organization

IUH = Indiana University Health healthcare system

IUHP = Indiana University Health Physicians employer of the EM physicians and APPs in this study

DEM = Department of Emergency Medicine at Indiana University School of Medicine is the academic department for the EM physicians in this study

PPE = Personal Protective Equipment (includes face masks, shields, respirators, gowns, gloves, eye protection)
