# Supplementary material for: Longitudinal Prospective Study of Emergency Medicine Provider Wellness Across Ten Academic and Community Hospitals During the Initial Surge of the COVID-19 Pandemic
Source: Res Sq. 2020 Oct 15:rs.3.rs-87786. Preprint. [Version 1] doi: 10.21203/rs.3.rs-87786/v1 (PMC7574355; doi:10.21203/rs.3.rs-87786/v1)
Supplement: Supplement [file 684a7638a5900510ef5c31d7.docx]

| **Supplemental Table 3: Emergency Medicine Provider Wellness Survey Responses – Identified Needs** | | | | | | |
| --- | --- | --- | --- | --- | --- | --- |
| **Question:**  “Yes” Responses No. (%) | **Week 1**  **3/30 – 4/6/20**  No. = 113 | **Week 2**  **4/7 – 4/12/20**  No. = 88^1^ | **Week 3**  **4/13 – 4/20/20**  No. = 75^1^ | **Week 4**  **4/21 – 4/27/20**  No. = 65^1^ | **p-value^2^**  **across all weeks** | **p-value^2^**  **week 1 vs. week 4** |
| **Provider type^3^** |  |  |  |  |  |  |
| - Physician | 84 (74%) | 64 (73%) | 57 (76%) | 49 (75%) |  |  |
| - APP | 29 (26%) | 24 (27%) | 18 (24%) | 16 (25%) |  |  |
| **I am concerned about my personal safety and/or the safety of family and dependents due to COVID-19 this week.** | **96 (85%)** | **66 (75%)** | **48 (64%)** | **40 (61%)** | 0.001 | <0.001 |
| - Physician | 73 (87%) | 46 (72%) | 37 (65%) | 31 (63%) |  |  |
| - APP | 23 (79%) | 20 (83%) | 11 (61%) | 9 (56%) |  |  |
| Interventions that would make you feel safer:^4^ |  |  |  |  |  |  |
| - Additional PPE | 85 (89%) | 37 (56%) | 25 (33%) | 21 (32%) |  |  |
| - Hospital-provided scrubs | 60 (63%) | 30 (45%) | 17 (23%) | 9 (14%) |  |  |
| - Shower at work | 46 (48%) | 22 (33%) | 15 (20%) | 5 (8%) |  |  |
| **My basic self-care (sleep, hygiene, nutrition, exercise) has been impacted this week.** | **75 (66%)** | **48 (55%)** | **32 (43%)** | **21 (32%)** | <0.001 | <0.001 |
| - Physician | 58 (69%) | 37 (58%) | 25 (44%) | 13 (27%) |  |  |
| - APP | 17 (59%) | 11 (46%) | 7 (39%) | 8 (50%) |  |  |
| Resources that would mitigate the impact on your self-care: ^4^ |  |  |  |  |  |  |
| - Help obtaining groceries | 13 (17%) | 11 (23%) | 2 (4%) | 2 (3%) |  |  |
| - A place to sleep near work | 12 (16%) | 3 (6%) | 2 (3%) | 3 (5%) |  |  |
| - Shower at work | 28 (37%) | 14 (29%) | 6 (8%) | 2 (3%) |  |  |
| - Gym/Exercise resources | 4 (5%) | - | 1 (1%) | 1 (1%) |  |  |
| - Nutrition resources | 3 (4%) | 2 (4%) | 1 (1%) | - |  |  |
| **The ability to care for my children, dependents, and/or pets has been impacted this week.** | **32 (29%)** | **20 (23%)** | **12 (16%)** | **13 (20%)** | 0.22 | 0.21 |
| - Physician | 24 (29%) | 18 (28%) | 11 (19%) | 12 (25%) |  |  |
| - APP | 8 (29%) | 2 (8%) | 1 (6%) | 1 (6%) |  |  |
| I need help getting dependent care resources. ^4^ | 3 (9%) | 1 (5%) | 1 (9%) | - | 0.79 | 0.55 |
| **I have experienced stress, anxiety, or fear due to COVID19 this week.** | **94 (83%)** | **64 (73%)** | **54 (72%)** | **43 (66%)** | 0.06 | 0.009 |
| - Physician | 70 (83%) | 46 (72%) | 43 (75%) | 33 (67%) |  |  |
| - APP | 24 (83%) | 18 (75%) | 11 (61%) | 10 (63%) |  |  |
| I have a mentor, colleague, friend, or family member to help me decompress. ^4^ | 84 (90%) | 50 (81%) | 44 (81%) | 40 (93%) | 0.13 | 0.75 |
| - Physician | 62 (89%) | 37 (80%) | 35 (81%) | 30 (91%) |  |  |
| - APP | 22 (96%) | 13 (81%) | 9 (82%) | 10 (100%) |  |  |
| I need mental health resources. ^4^ | 11 (12%) | 3 (5%) | 2 (4%) | 2 (5%) | 0.25 | 0.23 |
| - Physician | 8 (11%) | 1 (2%) | 2 (5%) | 2 (6%) |  |  |
| - APP | 3 (13%) | 2 (12%) | - | - |  |  |
| I need stress reduction resources. ^4^ | 28 (30%) | 8 (13%) | 6 (11%) | 4 (9%) | 0.005 | 0.009 |
| - Physician | 20 (29%) | 5 (11%) | 3 (7%) | 2 (6%) |  |  |
| - APP | 8 (33%) | 3 (18%) | 3 (27%) | 2 (20%) |  |  |
| **I have experienced strain on my relationships (partner, children, co-workers) due to COVID-19 this week.** | **57 (51%)** | **42 (48%)** | **33 (44%)** | **23 (35%)** | 0.26 | 0.05 |
| - Physician | 44 (52%) | 31 (48%) | 27 (48%) | 19 (39%) |  |  |
| - APP | 13 (45%) | 11 (46%) | 6 (33%) | 4 (25%) |  |  |
| **I have experienced personal illness or illness of a loved one this week.** | **23 (20%)** | **15 (17%)** | **8 (11%)** | **7 (11%)** | 0.20 | 0.10 |
| - Physician | 15 (18%) | 12 (19%) | 6 (11%) | 4 (8%) |  |  |
| - APP | 8 (28%) | 3 (13%) | 2 (11%) | 3 (19%) |  |  |
| Have you been quarantined this week? ^4^ | 9 (39%) | 6 (40%) | 1 (12%) | 1 (14%) | 0.38 | 0.37 |
| - Physician | 6 (40%) | 6 (50%) | 1 (17%) | 1 (25%) |  |  |
| - APP | 3 (38%) | - | - | - |  |  |
| Has one of your household contacts been quarantined this week? ^4^ | 2 (9%) | 2 (13%) | - | 1 (14%) | 0.78 | >0.99 |
| - Physician | 1 (7%) | 2 (17%) | - | 1 (25%) |  |  |
| - APP | 1 (13%) | - | - | - |  |  |
| **I have experienced additional work responsibilities or hours due to COVID-19 this week.** | **67 (59%)** | **43 (49%)** | **29 (39%)** | **22 (34%)** | 0.004 | 0.001 |
| - Physician | 52 (62%) | 36 (56%) | 23 (40%) | 18 (37%) |  |  |
| - APP | 15 (52%) | 7 (29%) | 6 (33%) | 4 (25%) |  |  |
| **I need shift coverage.** | **6 (5%)** | **2 (2%)** | **1 (1%)** | **1 (2%)** | 0.47 | 0.43 |
| - Physician | 5 (6%) | 2 (3%) | - | - |  |  |
| - APP | 1 (3%) | - | 1 (6%) | 1(6%) |  |  |
| **I have experienced loss of academic/scholarly productivity due to COVID-19 this week.** | **43 (38%)** | **27 (31%)** | **17 (23%)** | **18 (28%)** | 0.14 | 0.16 |
| - Physician | 40 (48%) | 27 (42%) | 17 (30%) | 16 (33%) |  |  |
| - APP | 3 (10%) | - | - | 2 (13%) |  |  |
| **I have experienced feelings of isolation due to COVID-19 this week.** | **64 (57%)** | **54 (61%)** | **46 (61%)** | **42 (65%)** | 0.75 | 0.30 |
| - Physician | 49 (58%) | 39 (61%) | 36 (63%) | 32 (65%) |  |  |
| - APP | 15 (52%) | 15 (63%) | 10 (56%) | 10 (63%) |  |  |
| I need resources to combat social isolation. ^4^ | 7 (11%) | 5 (9%) | 5 (11%) | 3 (7%) | 0.95 | 0.74 |
| - Physician | 6 (12%) | 3 (8%) | 2 (6%) | 2 (6%) |  |  |
| - APP | 1 (7%) | 2 (14%) | 3 (30%) | 1 (10%) |  |  |
| **I feel supported by my leadership.** | **100 (89%)** | **82 (93%)** | **74 (99%)** | **64 (98%)** | 0.01 | 0.02 |
| - Physician | 72 (86%) | 58 (91%) | 56 (98%) | 48 (98%) |  |  |
| - APP | 28 (97%) | 24 (100%) | 18 (100%) | 16 (100%) |  |  |
| I need more support from hospital leadership. ^4^ | 12 (92%) | 5 (83%) | 1 (100%) | - | 0.29 | 0.14 |
| - Physician | 11 (92%) | 5 (83%) | 1 (100%) | - |  |  |
| - APP | 1 (100%) | - | - | - |  |  |
| I need more support from department leadership. ^4^ | 8 (62%0 | 4 (67%) | 1 (100%) | 1 (100%) | >0.99 | >0.99 |
| - Physician | 7 (58%) | 4 (67%) | 1 (100%) | 1 (100%) |  |  |
| - APP | 1 (100%) | - | - | - |  |  |

^1^Total No. of respondents completing survey questions. Frequency of missing responses was 5 in week 2, 1 in week 2, and 1 in week 4.

^2^p-value determined by either Chi-squared or Fisher’s Exact where appropriate

^3^Physician and APP totals used as denominator to calculate percentages for each group in the table.

^4^Branching question, No. of respondents is less than total reported for the week.
