# Supplementary material for: Longitudinal Prospective Study of Emergency Medicine Provider Wellness Across Ten Academic and Community Hospitals During the Initial Surge of the COVID-19 Pandemic
Source: Res Sq. 2020 Oct 15:rs.3.rs-87786. Preprint. [Version 1] doi: 10.21203/rs.3.rs-87786/v1 (PMC7574355; doi:10.21203/rs.3.rs-87786/v1)
Supplement: Supplement [file 526860ae918ec3e78e5c9f19.docx]

**Supplemental Table 2: Occupational Characteristics of Non-Respondents**

|  | **Occupation** | | | | | | | |
| --- | --- | --- | --- | --- | --- | --- | --- | --- |
|  | **Week 1** | | **Week 2** | | **Week 3** | | **Week 4** | |
|  | **Physician** | **APP^2^** | **Physician** | **APP^2^** | **Physician** | **APP^2^** | **Physician** | **APP^2^** |
| **No. (%)^1^** | 73 (46) | 27 (48) | 92 (59) | 28 (50) | 99 (63) | 38 (68) | 107 (68) | 40 (71) |

^1^Eligible participants = 157 physicians and 56 APPs.

^2^APP = Advanced Practice Provider (nurse practitioner or physician assistant)
