# Supplementary material for: Longitudinal Prospective Study of Emergency Medicine Provider Wellness Across Ten Academic and Community Hospitals During the Initial Surge of the COVID-19 Pandemic
Source: Res Sq. 2020 Oct 15:rs.3.rs-87786. Preprint. [Version 1] doi: 10.21203/rs.3.rs-87786/v1 (PMC7574355; doi:10.21203/rs.3.rs-87786/v1)
Supplement: Supplement [file 4722bcb4a30bec3a2a6cf90f.pdf]

COVID Wellness Survey

This survey is VOLUNTARY and ANONYMOUS.

The EM wellness survey team wants to know about the wellness issues you are facing during the COVID-19 pandemic. We ask that you complete a series of anonymous surveys over the next few weeks. They take 3 mins or less of your time. Our goal is to identify issues and advocate for changes to improve frontline provider wellness.

Collected data is meant to assess needs of our EM providers and improve resources on the departmental, not individual level. Data is managed by the study team and will only be analyzed by in aggregate form to minimize any potential for identification of individual providers. Taking the survey demonstrates consent. There is no penalty for not participating. Please contact the study team for more information.

Start of Block: Demographics

Q1 Role

____

Display This Question:

If If Role Text Response Is Equal to Resident

Q2 Level of Training

____

Q3 Current FTE

____

Q4 Primary Work Site

____

Display This Question:

If If Primary Work Site Text Response Is Equal to Multiple Sites

Q5 Please select all that apply

____

Q6 Other Work Site

____

Q7 Years since completion of training

____

Q8 Sex

____

Q9 Age

____

End of Block: Demographics

Q10 Please identify the specific ways your life has been impacted by the COVID-19 pandemic in the past week.

Q11 Concerns about my personal safety and/or the safety of family and dependents due to COVID-19 this week.

- ◯ Yes (1)
- ◯ No (2)

Display This Question:

If Concerns about my personal safety and/or the safety of family and dependents due to COVID-19 this... = Yes

Q12 Please select the interventions that would make you feel safer at work:

- ▢ Additional PPE (1)
- ▢ Clean, hospital-provided scrubs (2)
- ▢ A place to shower after my shift (3)
- ▢ Other (4)

Display This Question:

If Please select the interventions that would make you feel safer at work: = Other

Q13 If other, please list:

____

Q14 Basic self care (sleep, hygiene, nutrition, exercise) has been impacted this week.

- ○ Yes (1)
- ○ No (2)

Display This Question:

If Basic self care (sleep, hygiene, nutrition, exercise) has been impacted this week. = Yes

Q15 Check all those that would mitigate the impact on your basic self care

- ☐ Help obtaining groceries or toiletries (1)
- ☐ A place to sleep at work or near work (2)
- ☐ A place to shower after a shift (3)
- ☐ Other (4)

Display This Question:

If Check all those that would mitigate the impact on your basic self care = Other

Q16 If other, please list:

____

Q17 Ability to care for my children, dependents, and/or pets has been impacted this week.

- ○ Yes (1)
- ○ No (2)

Display This Question:

If Ability to care for my children, dependents, and/or pets has been impacted this week. = Yes

Q18 I need help getting dependent care resources.

- ○ Yes (1)
- ○ No (2)

Q19 I have experienced stress, anxiety, or fear due to COVID-19 this week.

- ○ Yes (1)
- ○ No (2)

Display This Question:

If I have experienced stress, anxiety, or fear due to COVID-19 this week. = Yes

Q20 I have a mentor, colleague, friend, or family member to help me decompress.

- ○ Yes (4)
- ○ No (5)

Display This Question:

If I have experienced stress, anxiety, or fear due to COVID-19 this week. = Yes

Q21 I need mental health resources.

- ○ Yes (4)
- ○ No (5)

Display This Question:

If I have experienced stress, anxiety, or fear due to COVID-19 this week. = Yes

Q22 I need stress-reduction resources.

- ○ Yes (4)
- ○ No (5)

Q23 Strain on my relationships (partner, children, co-workers) due to COVID-19 this week.

○ Yes (1)

○ No (2)

Q24 Personal illness or illness of a loved one this week.

○ Yes (1)

○ No (2)

Display This Question:

If Personal illness or illness of a loved one this week. = Yes

Q25 Have you been quarantined?

○ Yes (4)

○ No (5)

Display This Question:

If Personal illness or illness of a loved one this week. = Yes

Q26 Has one of your household contacts been quarantined?

○ Yes (4)

○ No (5)

Q27 Additional work responsibilities or hours this week due to COVID-19.

- Yes (1)
- No (2)

Q28 I need shift coverage.

- Yes (1)
- No (2)

Q29 Loss of academic/scholarly productivity due to COVID-19 this week.

- Yes (1)
- No (2)

Q30 Feelings of isolation due to COVID-19 this week.

- Yes (1)
- No (2)

Display This Question:

If Feelings of isolation due to COVID-19 this week. = Yes

Q31 I need resources to combat social isolation.

- Yes (1)
- No (2)

Q32 I feel supported by my leadership.

◯ Yes (1)

◯ No (2)

Display This Question:

If I feel supported by my leadership. = No

Q33 I need more support from hospital leadership.

◯ Yes (4)

◯ No (5)

Display This Question:

If I feel supported by my leadership. = No

Q34 I need more support from department leadership.

◯ Yes (1)

◯ No (2)

End of Block: COVID Impact

Start of Block: Well-Being Index

Q35 The questions below relate to provider wellness.

Q36 During the past month, have you felt burned out from your work?

◯ Yes (4)

◯ No (5)

Q37 During the past month, have you worried that your work is hardening you emotionally?

- Yes (7)
- No (8)

Q38 During the past month, have you often been bothered by feeling down, depressed, or hopeless?

- Yes (4)
- No (5)

Q39 During the past month, have you fallen asleep while sitting inactive in a public place?

- Yes (4)
- No (5)

Q40 During the past month, have you felt that all the things you had to do were piling up so high that you could not overcome them?

- Yes (1)
- No (2)

Q41 During the past month, have you been bothered by emotional problems (such as feeling anxious, depressed, or irritable)?

- Yes (4)
- No (5)

Q42 During the past month, has your physical health interfered with your ability to do your daily work at home and/or away from home?

- ○ Yes (7)
- ○ No (8)

Q43 Please rate your level of agreement with the following statement: The work I do is meaningful to me.

- ○ Very Strongly Agree (4)
- ○ Strongly Agree (5)
- ○ Agree (6)
- ○ Neutral (7)
- ○ Disagree (8)
- ○ Strongly Disagree (9)
- ○ Very Strongly Disagree (10)

Q44 Please rate your level of agreement with the following statement: My work schedule leaves me enough time for my personal/family life.

- ○ Strongly agree (12)
- ○ Agree (13)
- ○ Neutral (14)
- ○ Disagree (15)
- ○ Strongly disagree (16)

End of Block: Well-Being Index

Start of Block: Brief Resilience Scale

Q45 Please rate the following statements.

	Strongly agree (13)	Agree (14)	Neutral (15)	Disagree (16)	Strongly disagree (17)
I tend to bounce back quickly after hard times. (1)	○	○	○	○	○
I have a hard time making it through stressful events. (2)	○	○	○	○	○
It does not take me long to recover from a stressful event. (3)	○	○	○	○	○
It is hard for me to snap back when something bad happens. (4)	○	○	○	○	○
I usually come through difficult times with little trouble. (5)	○	○	○	○	○
I tend to take a long time to get over set-backs in my life. (20)	○	○	○	○	○

End of Block: Brief Resilience Scale

Start of Block: Single Q and text box

Q46 Overall, based on your definition of burnout, how would your rate your level of burnout?

○ I enjoy my work. I have no symptoms of burnout. (1)

○ I am under stress, and I don't always have as much energy as I once did, but I don't feel burned out. (4)

○ I am definitely burning out and have one or more symptoms of burnout, such as physical or emotional exhaustion. (5)

○ The symptoms of burnout that I'm experiencing won't go away. I think about frustration at work a lot. (6)

○ I feel completely burned out and often wonder if I can go on. I am at the point where I may need some changes or may need to seek some sort of help. (7)

Q47 Please list the #1 issue affecting your wellness. Also provide any additional comments, ideas, or improvements that could be made to improve wellness.

____

End of Block: Single Q and text box
